# A Substructure Condensed Approach for Kinetostatic Modeling of Compliant Mechanisms with Complex Topology

**DOI:** 10.3390/mi13101734

**Published:** 2022-10-13

**Authors:** Shilei Wu, Zhongxi Shao, Hongya Fu

**Affiliations:** 1 School of Mechanical Engineering, Suzhou University of Science and Technology, Suzhou 215009, China; 2 School of Mechatronics Engineering, Harbin Institute of Technology, 92 West Dazhi Street, Harbin 150001, China

**Keywords:** compliant mechanisms, flexible elements, element transfer matrix, transfer matrix approach

## Abstract

Compliant mechanisms with complex topology have previously been employed in various precision devices due to the superiorities of high precision and compact size. In this paper, a substructure condensed approach for kinetostatic analysis of complex compliant mechanisms is proposed to provide concise solutions. In detail, the explicit relationships between the theoretical stiffness matrix, element stiffness matrix, and element transfer matrix for the common flexible beam element are first derived based on the energy conservation law. The transfer matrices for three types of serial–parallel substructures are then developed by combining the equilibrium equations of nodal forces with the transfer matrix approach, so that each branch chain can be condensed into an equivalent beam element. Based on the derived three types of transfer matrices, a kinetostatic model describing only the force-displacement relationship of the input/output nodes is established. Finally, two typical precision positioning platforms with complex topology are employed to demonstrate the conciseness and efficiency of this modeling approach. The superiority of this modeling approach is that the input/output stiffness, coupling stiffness, and input/output displacement relations of compliant mechanisms with multiple actuation forces and complex substructures can be simultaneously obtained in concise and explicit matrix forms, which is distinct from the traditional compliance matrix approach.

## 1. Introduction

With the superiorities of high precision, no clearance, no friction, and no assembly [1,2,3,4], compliant mechanisms have been increasingly employed in the fields of precision positioning [5,6,7,8], precision machining [9,10,11,12], and micro-electro mechanical systems (MEMS) [13,14], and so forth. Compared with conventional rigid-body mechanisms, compliant mechanisms convert energy, forces, and motion using the elastic deformations of flexible elements. Both kinematic and elasto-mechanical behaviors need to be considered simultaneously. Therefore, the design and analysis of compliant mechanisms are much more complicated and labor intensive than those of conventional rigid-body mechanisms.

In order to accurately analyze compliant mechanisms, it is crucial to establish a concise and efficient kinetostatic model for performance analysis, optimization design, and motion control. Finite element analysis is a common and effective approach for the kinetostatic analysis of compliant mechanisms with arbitrarily complex topology. In this approach, compliant mechanisms are first discretized into a large number of flexible elements. The kinetostatic model can then be derived based on the topology relationships of the flexible elements [15,16]. However, it fails to provide a concise and explicit motion control model and is also inapplicable for fast performance prediction due to the considerable degrees of freedom of the model. Hence, a number of studies focusing on this area have been carried out over the past few decades.

To develop the kinetostatic models for compliant mechanisms with small deflections, numerous modeling approaches have been proposed, for instance, the elastic beam theory [17,18], Castigliano’s second theorem [19,20], the compliance matrix approach [21,22,23], and the transfer matrix approach [24,25,26]. According to the literatures, the elastic beam theory and Castigliano’s second theorem are the most common ways for kinematic and static analyses of various flexure hinges and compliant mechanisms with relatively simple topology. Ma [27] simplified the parallel-type compliant amplifier into a rhombic-type amplifier and developed the analytical kinetostatic model for this amplifier by employing the elastic beam theory. Subsequently, the elastic beam theory was increasingly applied in the design and analysis of amplifying mechanisms. Lobontiu et al. [28,29] derived the stiffness models of various flexure hinges based on Castigliano’s second theorem. Moreover, the theoretical models of input stiffness, amplification ratio, and the output stiffness of bridge-type amplifying mechanisms were established by this approach in [19]. However, the modeling approaches based on the elastic beam theory or Castigliano’s second theorem require tedious internal force analysis, which is inapplicable for compliant mechanisms with complex topology. Compared with those two approaches, the compliance matrix approach can avoid the analysis of tedious internal forces. Therefore, the compliance matrix approach is particularly suitable for kinetostatic analysis of compliant mechanisms with complex topology. It was used to develop the stiffness models for compliant mechanisms with series and parallel topology in [30]. Additionally, Li and Xu [31,32] applied the compliance matrix approach to derive the analytical kinetostatic models for various compliant precision positioning manipulators. It is worth mentioning that the forces and corresponding displacements are at the same point. Lobontiu [33,34] established a series of kinetostatic models of serial–parallel compliant mechanisms with complex conditions, such as multi-point force loads and complex branched substructures, by employing the compliance matrix approach. However, the output stiffness, input stiffness, and coupling stiffness need to be respectively modeled [16]. As a result, the kinetostatic modeling procedure will become complicated for complex serial–parallel topology. In order to resolve this problem, a matrix displacement approach based on the nodal forces equilibrium equations was proposed to establish the kinetostatic models of compliant mechanisms with serial–parallel topology [24]. To avoid the solution of labor intensive equilibrium equations of nodal forces, an energy-based approach was proposed in our previous research [35]. However, this modeling approach is still cumbersome and has a large number of elements. In view of this, a two-port dynamic stiffness model for complex serial–parallel topology was developed based on the transfer matrix approach [25]. It is worth mentioning that this modeling approach is only suitable for specific topology and the element dynamic stiffness matrix is very difficult to formulate.

As a result, it is still necessary to further improve the conciseness and accuracy of the kinetostatic models for complex serial–parallel compliant mechanisms. This paper aims to extend our previous research [35] to develop a concise and efficient kinetostatic model with a low number of elements for compliant mechanisms with complex topology. The novelty of this paper is to propose a substructure condensed approach for the kinetostatic modeling of compliant mechanisms with complex topology and realize the explicit expression of input/output displacement relations with concise and matrix forms. The major contributions of this paper are summarized as follows. The explicit relationships between the theoretical stiffness matrix, element stiffness matrix, and element transfer matrix of the common flexible beam element are established via the energy conservation law. Similarly, the transfer matrices for three types of serial–parallel substructures are developed by combining the equilibrium equations of nodal forces with the transfer matrix approach. Additionally, kinematic and static performance analyses such as input/output stiffness, coupling stiffness, and the input/output displacement relation of complex compliant mechanisms can be simultaneously obtained.

This paper is organized as follows. Section 2 presents the general expression of the element transfer matrix for the common flexible beam element derived by employing the energy conservation law. The general kinetostatic model describing the force-displacement relationships of the input/output nodes for complex serial–parallel topology is proposed by combining the equilibrium equations of nodal forces with the transfer matrix approach in Section 3. Two typical precision positioning platforms are employed to validate the conciseness and accuracy of the proposed modeling approach in Section 4. Finally, conclusions are drawn in Section 5.

## 2. General Expression of Element Transfer Matrix for the Common Flexible Beam Element

**Figure 1:**
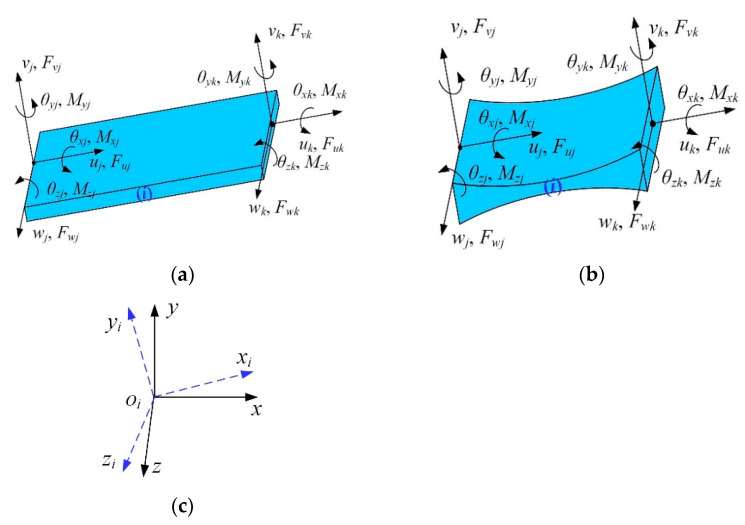
Nodal displacements and nodal forces of flexible elements: (**a**) the flexible beam with equal-section; (**b**) the flexible beam with variable-section; (**c**) the local and reference coordinate systems.

As the basic element of compliant mechanisms, the compliance matrices of flexible beams with equal-section or variable-section have been extensively studied. For example, the compliance matrices of sheet, elliptic, and hyperbolic flexure hinges have been established based on the elastic beam theory and Castigliano’s second theorem. As for flexible beams with equal-section, the element stiffness matrices can be calculated by employing the finite element approach. However, further investigation focusing on the general expression of the element transfer matrix for the common flexible element still needs to be carried out. As shown in Figure 1, the flexible beam element (*i*) has node *j* and node *k*, with six degrees of freedom per node. The force-displacement relationship of node *j* and node *k* in the local coordinate system is then obtained based on the elastic beam theory:

(1) $$\left\{\begin{array}{c} F_{i,j}^{e}\\ F_{i,k}^{e} \end{array}\right\}=K_{i}^{e}\left\{\begin{array}{c} x_{i,j}^{e}\\ x_{i,k}^{e} \end{array}\right\}$$

where $F_{i,j}^{e}={\left[F_{uj},F_{vj},F_{wj},M_{xj},M_{yj},M_{zj}\right]}^{T}$ and $F_{i,k}^{e}={\left[F_{uk},F_{vk},F_{wk},M_{xk},M_{yk},M_{zk}\right]}^{T}$ are, respectively, the nodal forces at the free end of the flexible beam element (*i*). $x_{i,j}^{e}={\left[u_{j},v_{j},w_{j},\theta_{xj},\theta_{yj},\theta_{zj}\right]}^{T}$ and $x_{i,k}^{e}={\left[u_{k},v_{k},w_{k},\theta_{xk},\theta_{yk},\theta_{zk}\right]}^{T}$ are the nodal displacements at the free the end of the flexible beam element (*i*). $K_{i}^{e}$ is the 12 × 12 element stiffness matrix in the local coordinate system.

Rewriting the above equations into a block submatrices form, Equation (1) can be further expressed as:

(2) $$\left\{\begin{array}{c} F_{i,j}^{e}\\ F_{i,k}^{e} \end{array}\right\}=\left[\begin{array}{cc} K_{i,1}^{e} & K_{i,2}^{e}\\ K_{i,3}^{e} & K_{i,4}^{e} \end{array}\right]\left\{\begin{array}{c} x_{i,j}^{e}\\ x_{i,k}^{e} \end{array}\right\}$$

where $K_{i,1}^{e}$, $K_{i,2}^{e}$, $K_{i,3}^{e}$, and $K_{i,4}^{e}$ are block submatrices of the element stiffness matrix in the local coordinate system.

This element can be seen as a flexible beam when the left end of the flexible beam element is clamped. The force-displacement relationship of node *k* can be obtained from Equation (2).

(3) $$F_{i,k}^{e}=K_{i,4}^{e}x_{i,k}^{e}=K_{i}x_{i,k}^{e}$$

where $K_{i}=K_{i,4}^{e}$ is the theoretical stiffness matrix of this flexible beam.

Equation (3) can be further rewritten as below:

(4) $$x_{i,k}^{e}=C_{i}F_{i,k}^{e}$$

where $C_{i}=K_{i}{}^{-1}$ is the theoretical compliance matrix of the flexible beam. The detailed expression of the compliance matrix can be derived from previous investigations [36,37] and expressed as below:

(5) $$C_{i}=\left[\begin{array}{cccccc} C_{u-F_{u}} & 0 & 0 & 0 & 0 & 0\\ 0 & C_{v-F_{v}} & 0 & 0 & 0 & C_{v-M_{z}}\\ 0 & 0 & C_{w-F_{w}} & 0 & C_{w-M_{y}} & 0\\ 0 & 0 & 0 & C_{\theta_{x}-M_{x}} & 0 & 0\\ 0 & 0 & C_{\theta_{y}-F_{w}} & 0 & C_{\theta_{y}-M_{y}} & 0\\ 0 & C_{\theta_{z}-F_{v}} & 0 & 0 & 0 & C_{\theta_{z}-M_{z}} \end{array}\right]$$

For the flexible beam element (*i*), the displacement at node *k* equals the linear superposition of the elastic deformation under the external force and the displacement at node *j*. Hence, the nodal displacement of node *k* can be expressed by Equation (6).

(6) $$x_{i,k}^{e}=\Delta x_{i}^{e}+[Ad]x_{i,j}^{e}$$

where $\Delta x_{i}^{e}$ is the elastic deformation of the flexible beam element (*i*). [Ad] is a 6 × 6 coordinate transformation matrix, which can transfer the nodal displacement at node *j* from the coordinate system *ox_j_y_j_z_j_* to the coordinate system *ox_k_y_k_z_k_*, as shown in Figure 1, and can be expressed as:

(7) $$[Ad]=\left[\begin{array}{cc} R & D(d)R\\ 0 & R \end{array}\right]$$

where ***R*** is a 3 × 3 rotation transformation matrix of the coordinate system *ox_j_y_j_z_j_* to the coordinate system *ox_k_y_k_z_k_*. ***d*** = [*d_x_*, *d_y_*, *d_z_*]^T^ is the position vector of node *j* expressed in the coordinate system *ox_k_y_k_z_k_*. ***D*** (***d***) is a skew-symmetric matrix of the position vector ***d*** and can be obtained as:

(8) $$D(d)=\left[\begin{array}{ccc} 0 & -d_{z} & d_{y}\\ d_{z} & 0 & -d_{x}\\ -d_{y} & d_{x} & 0 \end{array}\right]$$

The flexible element can be considered as a cantilever beam when the left end is clamped while the right end is free, and the elastic deformation at the free end is $\Delta x_{i}^{e}$. Based on the energy conservation law, the potential strain energy stored in the flexible beam element is equal to the potential strain energy stored in the cantilever beam. Therefore, the potential strain energy stored in the flexible beam element (*i*) can be derived as below:

(9) $$\begin{array}{ll} U_{i} & =\frac{1}{2}\Delta x_{i}^{eT}K_{i}\Delta x_{i}^{e}=\frac{1}{2}{\left(x_{i,k}^{e}-[Ad]x_{i,j}^{e}\right)}^{T}K_{i}(x_{i,k}^{e}-[Ad]x_{i,j}^{e})\\ & =\frac{1}{2}\left[\begin{array}{cc} x_{i,j}^{eT} & x_{i,k}^{eT} \end{array}\right]\left[\begin{array}{cc} {\left[Ad\right]}^{T}K_{i}[Ad] & -{\left[Ad\right]}^{T}K_{i}\\ -K_{i}[Ad] & K_{i} \end{array}\right]\left[\begin{array}{c} x_{i,j}^{e}\\ x_{i,k}^{e} \end{array}\right]\\ & =\frac{1}{2}x_{i}^{eT}K_{i}^{e}x_{i}^{e} \end{array}$$

The element stiffness matrix, $K_{i}^{e}$, of the flexible beam element (*i*) in the local coordinate system can then be obtained as below:

(10) $$K_{i}^{e}=\left[\begin{array}{cc} {\left[Ad\right]}^{T}K_{i}[Ad] & -{\left[Ad\right]}^{T}K_{i}\\ -K_{i}[Ad] & K_{i} \end{array}\right]$$

According to the above equation, Equation (1), which describes the force-displacement relationship of node *j* and node *k* in the local coordinate system, can be further given as follows:

(11) $$\left\{\begin{array}{c} F_{i,j}^{e}\\ F_{i,k}^{e} \end{array}\right\}=\left[\begin{array}{cc} {\left[Ad\right]}^{T}K_{i}[Ad] & -{\left[Ad\right]}^{T}K_{i}\\ -K_{i}[Ad] & K_{i} \end{array}\right]\left\{\begin{array}{c} x_{i,j}^{e}\\ x_{i,k}^{e} \end{array}\right\}$$

Rewriting Equation (11), the following relationship can be obtained:

(12) $$\left\{\begin{array}{c} x_{i,k}^{e}\\ F_{i,k}^{e} \end{array}\right\}=T_{i}^{e}\left\{\begin{array}{c} x_{i,j}^{e}\\ F_{i,j}^{e} \end{array}\right\}=\left[\begin{array}{cc} \left[Ad\right] & -K_{i}{}^{-1}{\left[Ad\right]}^{-T}\\ 0 & -{\left[Ad\right]}^{-T} \end{array}\right]\left\{\begin{array}{c} x_{i,j}^{e}\\ F_{i,j}^{e} \end{array}\right\}$$

where $T_{i}^{e}$ is the element transfer matrix of the flexible beam element (*i*) in the local coordinate system. The element transfer matrix can also describe the force-displacement relationship for two nodes at the free end in the local coordinate system.

**Figure 2:**
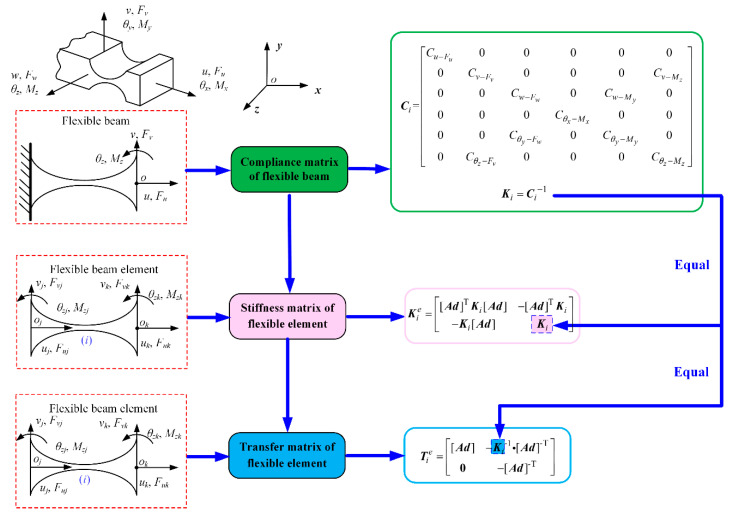
Transfer matrix of a flexible beam element.

Equations (11) and (12) establish the explicit relationships between theoretical stiffness matrix, element stiffness matrix, and element transfer matrix for the common flexible beam element. It can be observed that the element transfer matrix only depends on the coordinate transformation matrix [***Ad***] and the theoretical stiffness matrix ***K****_i_*. In order to reduce the degrees of freedom of the kinetostatic model, Ling [24] developed the kinetostatic model of serial–parallel compliant mechanisms by employing the transfer matrix approach. However, the author did not establish the explicit relationship between the theoretical stiffness matrix and the element transfer matrix. Compared with that approach, the general expression of the element transfer matrix with respect to the theoretical stiffness matrix for the common flexible element is established based on the energy conservation law. The explicit relationships between the theoretical stiffness matrix, the element stiffness matrix, and the element transfer matrix for the common flexible beam element are shown in Figure 2.

Due to this element stiffness matrix being developed in the local coordinate system, it needs to be converted into the reference coordinate system. This force-displacement relationship of node *j* and node *k* in the reference coordinate system is obtained as below:

(13) $$\left\{\begin{array}{c} x_{i,k}^{}\\ F_{i,k}^{} \end{array}\right\}=T_{i}^{}\left\{\begin{array}{c} x_{i,j}^{}\\ F_{i,j}^{} \end{array}\right\}$$

where $x_{i,j}^{}$, $x_{i,k}^{}$, and $F_{i,j}^{}$, $F_{i,k}^{}$ are the nodal displacements and nodal forces at two free ends in the reference coordinate system, respectively. ***T****_i_* is a 12 × 12 element transfer matrix in the reference coordinate system and can be calculated as:

(14) $$\left[T_{i}]={\left[Tr_{i}\right]}^{T}[T_{i}^{e}][Tr_{i}\right]$$

where ***Tr****_i_* is a 12 × 12 rotation transformation matrix, which can be expressed as below:

(15) $$Tr_{i}=\left[\begin{array}{cccc} R_{i} & 0 & 0 & 0\\ 0 & R_{i} & 0 & 0\\ 0 & 0 & R_{i} & 0\\ 0 & 0 & 0 & R_{i} \end{array}\right]$$

where ***R****_i_* is a 3 × 3 coordinate rotation matrix of the local coordinate system to the reference coordinate system.

## 3. Kinetostatic Modeling Based on the Substructure Condensed Approach

### 3.1. Transfer Matrices for Three Types of Substructures

**Figure 3:**
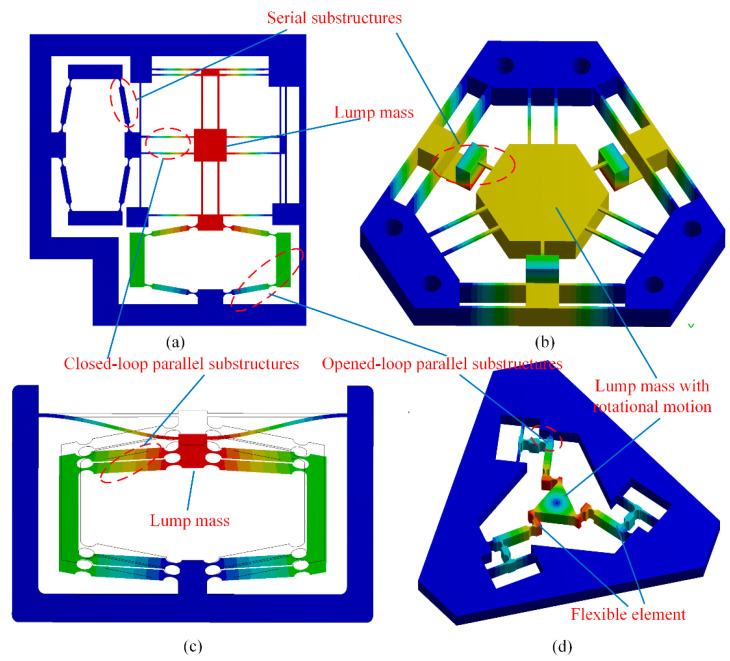
Typical compliant mechanisms: (**a**) a *XY* monolithic precision platform [35]; (**b**) a monolithic tip-tilt-piston spatial compliant manipulator [35]; (**c**) a one-dimensional precision positioning platform [38]; (**d**) a 3-DOF precision positioning platform [24,35].

**Figure 4:**
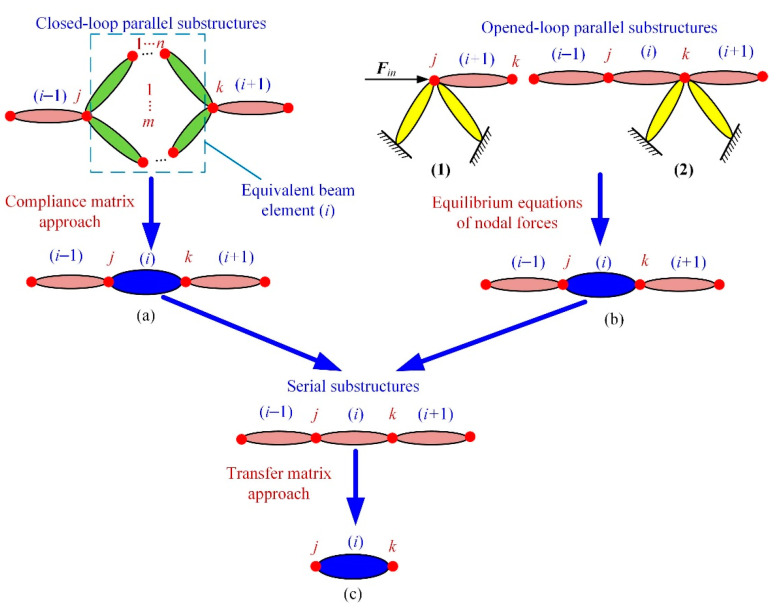
Illustration of serial and parallel substructures: (**a**) the closed-loop parallel substructure; (**b**) the opened-loop parallel substructure; (**c**) the series substructure.

In most cases, compliant mechanisms usually consist of flexible beams (equal-section flexible beam), flexure hinges (variable-section flexible beam), and lumped masses with series and/or parallel connections. For example, Figure 3 provides several typical compliant mechanisms with complex topology. The common characteristics of these compliant mechanisms are that the connection relationships can be divided into three types, namely series substructure, closed-loop parallel substructure, and opened-loop parallel substructure. Figure 4 illustrates the topology of these three types of connections abstracted from Figure 3.

For the closed-loop parallel substructure in Figure 4a, it can be condensed into an equivalent beam when the *j*-end of the equivalent beam element is clamped. This equivalent beam consists of *m* branches with a parallel connection where each branch consists of *n* flexible elements with a series connection. Based on the compliance matrix approach, the compliance matrix of the *t*-th parallel branch can be expressed as:

(16) $$C_{t}=\sum_{g=1}^{n}\left({\left[Ad\right]}_{g}K_{g}{}^{-1}{\left[Ad\right]}_{g}^{T}\right)$$

where ***K****_g_* is the theoretical stiffness matrix of the *g*-th flexible beam of the *t*-th parallel branch. [***Ad***]*_g_* is a 6 × 6 coordinate transformation matrix, which can be expressed by Equation (7).

In the same way, the theoretical stiffness matrix of the equivalent beam is obtained as follows:

(17) $$K_{i}={\sum_{t=1}^{m}\left({\left[Ad\right]}_{t}C_{t}{\left[Ad\right]}_{t}{}^{T}\right)}^{-1}$$

According to Equation (12), the element transfer matrix of the equivalent beam element (*i*) in the local coordinate system can be expressed as:

(18) $$T_{eq}^{e}=\left[\begin{array}{cc} \left[Ad\right] & -K_{i}{}^{-1}{\left[Ad\right]}^{-T}\\ 0 & -{\left[Ad\right]}^{-T} \end{array}\right]$$

By performing rotation transformation, the element transfer matrix ***T****_eq_* of the equivalent beam element (*i*) in the reference coordinate system can be obtained. The force-displacement relation of node *j* and node *k* of equivalent beam element (*i*) in the reference coordinate system can then be expressed as:

(19) $$\left\{\begin{array}{c} x_{i,k}^{}\\ F_{i,k}^{} \end{array}\right\}=T_{eq}^{}\left\{\begin{array}{c} x_{i,j}^{}\\ F_{i,j}^{} \end{array}\right\}$$

Therefore, the closed-loop parallel substructure can be condensed into an equivalent beam element by employing the compliance matrix approach.

For the opened-loop parallel substructure, the relationships between the nodal displacement/force at node *j* of flexible element (*i* + 1) and node *k* of flexible element (*i*) can be obtained based on the equilibrium equations of nodal forces.

(20) $$\left\{\begin{array}{l} \mathrm{Situations}\;1(\mathrm{input}\;\mathrm{node}):\left\{\begin{array}{l} x_{i+1,j}\\ F_{i+1,j} \end{array}\right\}=\left[\begin{array}{cc} E_{6\times 6} & 0_{6\times 6}\\ 0_{6\times 6} & -E_{6\times 6} \end{array}\right]\left[\begin{array}{cc} E_{6\times 6} & 0_{6\times 6}\\ \sum_{t=1}^{n}K_{i} & -E_{6\times 6} \end{array}\right]\left\{\begin{array}{l} x_{in}\\ F_{in} \end{array}\right\}=SQ_{in,i}\left\{\begin{array}{l} x_{in}\\ F_{in} \end{array}\right\}\\ \mathrm{Situations}\;2(\mathrm{other}\;\mathrm{node}):\left\{\begin{array}{l} x_{i+1,j}\\ F_{i+1,j} \end{array}\right\}=\left[\begin{array}{cc} E_{6\times 6} & 0_{6\times 6}\\ 0_{6\times 6} & -E_{6\times 6} \end{array}\right]\left[\begin{array}{cc} E_{6\times 6} & 0_{6\times 6}\\ \sum_{t=1}^{n}K_{i} & E_{6\times 6} \end{array}\right]\left\{\begin{array}{l} x_{i,k}\\ F_{i,k} \end{array}\right\}=SQ_{i}\left\{\begin{array}{l} x_{i,k}\\ F_{i,k} \end{array}\right\} \end{array}\right.$$

where ***E***_6×6_ is a 6 × 6 identity matrix and **0**_6×6_ is a 6 × 6 zero matrix. ***x****_in_* and ***F****_in_* are input displacement and input force, respectively. ***Q****_in_*_,*i*_ and ***Q****_i_* indicate the force summation. ***S*** is a transfer matrix, which can transfer the force and displacement at node *k* of flexible element (*i*) to node *j* of the flexible element (*i*).

Therefore, the parallel substructure, such as that in Figure 4a,b, can be condensed into an equivalent beam element based on the compliance matrix approach and nodal forces equilibrium equations.

As to the series substructure, the relationship between the nodal displacement/force at node *j* of the flexible element (*i* + 1) and at node *k* of the flexible element (*i*) can be obtained in a matrix form based on Newton’s third law.

(21) $$\left\{\begin{array}{l} u_{i+1,j}\\ F_{i+1,j} \end{array}\right\}=\left[\begin{array}{cc} E_{6\times 6} & 0_{6\times 6}\\ 0_{6\times 6} & -E_{6\times 6} \end{array}\right]\left\{\begin{array}{l} u_{i,k}\\ F_{i,k} \end{array}\right\}=S\left\{\begin{array}{l} u_{i,k}\\ F_{i,k} \end{array}\right\}$$

Substituting Equation (19) into Equation (21) and substituting Equation (13) into Equations (20) and (21), the relationship between the nodal displacement/force at node *j* of flexible element (*i* + 1) and at node *j* of flexible element (*i*) can be calculated as follows:

(22) $$\left\{\begin{array}{cc} \mathrm{Closed-loop parallel substructure}: & \left\{\begin{array}{c} u_{i+1,j}\\ F_{i+1,j} \end{array}\right\}=(ST_{eq})\left\{\begin{array}{l} u_{i,j}\\ F_{i,j} \end{array}\right\}\\ \mathrm{Opened-loop parallel substructure}\;1: & \left\{\begin{array}{c} u_{i+1,j}\\ F_{i+1,j} \end{array}\right\}=(SQ_{in,i})\left\{\begin{array}{l} u_{in}\\ F_{in} \end{array}\right\}\\ \mathrm{Opened-loop parallel substructure}\;2: & \left\{\begin{array}{l} u_{i+1,j}\\ F_{i+1,j} \end{array}\right\}=(SQ_{i}T_{i})\left\{\begin{array}{l} u_{i,j}\\ F_{i,j} \end{array}\right\}\\ \mathrm{Serise substructure}: & \left\{\begin{array}{l} u_{i+1,j}\\ F_{i+1,j} \end{array}\right\}=(ST_{i})\left\{\begin{array}{l} u_{i,j}\\ F_{i,j} \end{array}\right\} \end{array}\right.$$

### 3.2. Establishing Kinetostatic Model

**Figure 5:**
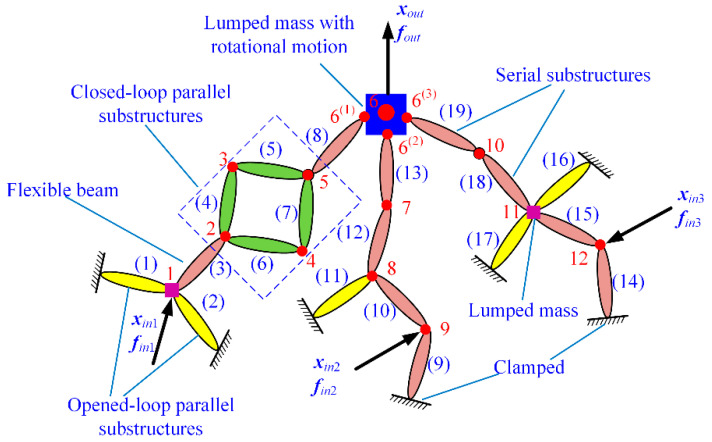
General topology of typical compliant mechanisms.

To better describe the proposed modeling approach, a general topology for typical compliant mechanisms with complex connections abstracted from Figure 3 is employed, as shown in Figure 5, which can represent most cases in applications. The compliant mechanism consists of three branch chains, each of which comprises flexible elements and lumped masses with series and/or parallel connections. The lumped mass with rotational motion is simplified to one node at the rotary center and three sub-nodes at the connection nodes with three branch chains, which is shown in Figure 5. The proposed modeling procedure consists of five steps:

#### 3.2.1. Discretizing and Numbering

The flexible beams of three branch chains are denoted from (1) to (19), and the connection nodes are numbered from 1 to 12, in which all clamped nodes are denoted as 0. The input forces are denoted as ***f****_in_*_1_, ***f****_in_*_2_, and ***f****_in_*_3_. The input displacements are denoted as ***x****_in_*_1_, ***x****_in_*_2_, and ***x****_in_*_3_. The output force (external force) and output displacement of the output platform are, respectively, numbered as ***f****_out_* and ***x****_out_*. These three sub-nodes are numbered as 6^(1)^, 6^(2)^, and 6^(3)^.

#### 3.2.2. Calculating Transfer Matrices of Flexible Beams and Lumped Mass

**Figure 6:**
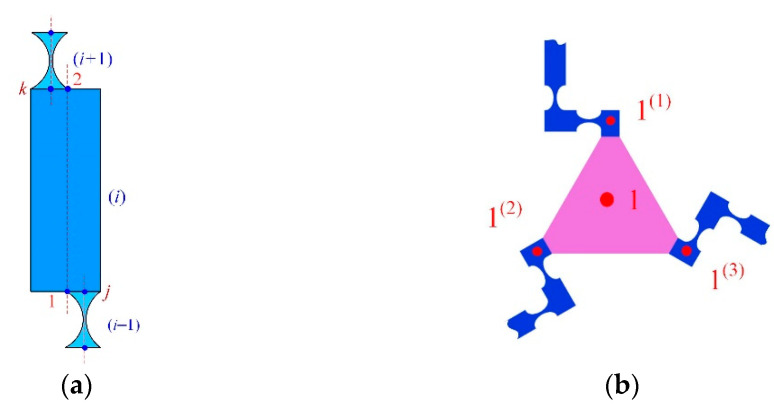
Nodal displacements and nodal forces: (**a**) the flexible beam; (**b**) the lumped mass.

For the common flexible element with nodes on the axis of symmetry, the element transfer matrix in the local coordinate system can be formulated using Equation (12). However, the flexible element whose nodes are not on the axis of symmetry, such as the flexible element in Figure 3d, are widely used in compliant mechanisms. Their element transfer matrix cannot be calculated directly based on Equation (12). As shown in Figure 6a, the nodal forces at node 1 and node 2 of the flexible element in regard to node *j* and node *k* can be obtained by performing the coordinate transformation matrix.

(23) $$F_{i,1}^{e}=[Ad_{1}{]}^{-T}F_{i,j}^{e},\;\;\begin{array}{c} F_{i,2}^{e}=[Ad_{2}{]}^{-T} \end{array}F_{i,k}^{e}$$

where [***Ad***_1_] and [***Ad***_2_] are coordinate transformation matrices from the coordinate systems *o_j_x_j_y_j_z_j_* and *o_k_x_k_y_k_z_k_* into the coordinate systems *o*_1_*x*_1_*y*_1_*z*_1_ and *o*_2_*x*_2_*y*_2_*z*_2_, respectively.

Similarly, the nodal displacements at node 1 and node 2 of the flexible element (*i*) in regard to node *j* and node *k* can be expressed as:

(24) $$x_{i,1}^{e}=[Ad_{1}]x_{i,j}^{e},\;\;\begin{array}{c} x_{i,2}^{e}=[Ad_{2}] \end{array}x_{i,k}^{e}$$

The force-displacement relationship of node 1 and node 2 of this element in the local coordinate system can then be obtained based on the elastic beam theory.

(25) $$\left\{\begin{array}{c} F_{i,1}^{e}\\ F_{i,2}^{e} \end{array}\right\}=\left[\begin{array}{cc} {\left[Ad\right]}^{T}K_{i}[Ad] & -{\left[Ad\right]}^{T}K_{i}\\ -K_{i}[Ad] & K_{i} \end{array}\right]\left\{\begin{array}{c} x_{i,1}^{e}\\ x_{i,2}^{e} \end{array}\right\}$$

By substituting Equations (23) and (24) into Equation (12), the force-displacement relationship of the flexible element (*i*) in regard to node *j* and node *k* can be transferred to:

(26) $$\left\{\begin{array}{c} x_{i,k}^{e}\\ F_{i,k}^{e} \end{array}\right\}=\left[\begin{array}{cc} {\left[Ad_{2}\right]}^{-1}[Ad][Ad_{1}] & -{\left[Ad_{2}\right]}^{-1}K_{i}{}^{-1}{\left[Ad\right]}^{-T}{\left[Ad_{1}\right]}^{-1}\\ 0 & -{\left[Ad_{2}\right]}^{T}{\left[Ad\right]}^{-T}{\left[Ad_{1}\right]}^{-T} \end{array}\right]\left\{\begin{array}{c} x_{i,j}^{e}\\ F_{i,j}^{e} \end{array}\right\}$$

For the lumped mass with only translational motion, such as the output platforms in Figure 3a,c, it can be regarded as a node. However, the lumped mass with rotational motion, such as the output platforms in Figure 3b,d, cannot be simply regarded as a node. As shown in Figure 6b, it is equivalent to one node at its rotary center and three sub-nodes at the connection nodes with three branch chains. It should be noted that the displacement of sub-node 1^(1)^, sub-node 1^(2)^, and sub-node 1^(3)^ is not equal to the displacement of node 1 due to the rotational motion. The nodal displacement and nodal force at the rotary center can be transferred to three sub-nodes at the connection nodes with three branch chains by performing a coordinate transformation matrix. The nodal displacement and nodal force of the lumped mass are then contained in the element transfer matrix of the adjacent flexible element. Therefore, the issue of the lumped mass with rotational motion in compliant mechanisms is solved.

#### 3.2.3. Calculating the Transfer Matrix of Each Branch Chain

The compliant mechanism consists of three branch chains, and each branch chain includes one input node and one output node. According to the three connection types, the transfer matrix of each branch chain from input node to output node can be calculated as:

(27) $$\left\{\begin{array}{l} T_{ch1}=(T_{8})(ST_{eq})(ST_{2})(SQ_{in,1})\\ T_{ch2}=(T_{13})(ST_{12})(SQ_{10}T_{10})(SQ_{in,2})\\ T_{ch3}=(T_{19})(ST_{18})(SQ_{15}T_{15})(SQ_{in,3}) \end{array}\right.$$

where ***T****_i_* and ***S*** are transfer matrices and given by Equations (12), (26), and (21). ***Q****_i_*, ***Q****_in_*_,1_, ***Q****_in_*_,2_, and ***Q****_in_*_,3_ are summation matrices, which can be expressed as:

(28) $$\left\{\begin{array}{l} Q_{in,1}=\left[\begin{array}{cc} E_{6\times 6} & 0_{6\times 6}\\ K_{1}+K_{2} & -E_{6\times 6} \end{array}\right],Q_{in,2}=\left[\begin{array}{cc} E_{6\times 6} & 0_{6\times 6}\\ K_{9} & -E_{6\times 6} \end{array}\right],Q_{in,3}=\left[\begin{array}{cc} E_{6\times 6} & 0_{6\times 6}\\ K_{14} & -E_{6\times 6} \end{array}\right]\\ Q_{10}=\left[\begin{array}{cc} E_{6\times 6} & 0_{6\times 6}\\ K_{11} & E_{6\times 6} \end{array}\right],Q_{15}=\left[\begin{array}{cc} E_{6\times 6} & 0_{6\times 6}\\ K_{16}+K_{17} & E_{6\times 6} \end{array}\right] \end{array}\right.$$

#### 3.2.4. Establishing the Kinetostatic Model of the Compliant Mechanism

**Figure 7:**
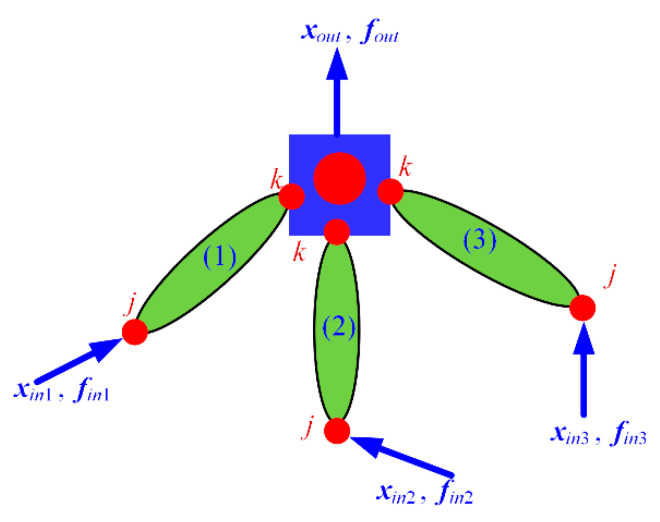
The equivalent topology of typical compliant mechanisms.

Through the above analysis, the general topology of the typical compliant mechanisms in Figure 5 can be condensed into the equivalent topology including only the input node and the output node, as shown in Figure 7. Based on the transfer matrices of three branch chains, the force-displacement relationships of three equivalent beam elements can be obtained as:

(29) $$\left\{\begin{array}{c} f_{i,j}\\ f_{i,k} \end{array}\right\}=\left[\begin{array}{cc} k_{i,1} & k_{i,2}\\ k_{i,3} & k_{i,4} \end{array}\right]\left\{\begin{array}{c} x_{in,j}\\ x_{i,k} \end{array}\right\}$$

where ***k****_i_*_,1_, ***k****_i_*_,2_, ***k****_i_*_,3_, and ***k****_i_*_,4_ (*i* = 1,2,3) are block sub-matrices and can be obtained from the transfer matrix of each branch chain, ***T****_chi_*.

Taking the input/output nodes as research objects, the input/output force-displacement relations of the compliant mechanism can be calculated based on the equilibrium equations of nodal forces.

(30) $$\left\{\begin{array}{l} f_{in1}=k_{1,1}x_{in1}+k_{1,2}x_{out}\\ f_{in2}=k_{2,1}x_{in2}+k_{2,2}x_{out}\\ f_{in3}=k_{3,1}x_{in3}+k_{3,2}x_{out}\\ f_{out}=k_{1,3}x_{1}+k_{2,3}x_{in2}+k_{3,3}x_{in3}+(k_{1,4}+k_{2,4}+k_{3,4})x_{out} \end{array}\right.$$

Rewriting the above equations into a matrix form, the following kinetostatic model of compliant mechanisms with complex topology can be expressed as:

(31) $$\left\{\begin{array}{l} f_{in1}\\ f_{in2}\\ f_{in3}\\ f_{out} \end{array}\right\}=\left[\begin{array}{cccc} k_{1,1} & 0 & 0 & k_{1,2}\\ 0 & k_{2,1} & 0 & k_{2,2}\\ 0 & 0 & k_{3,1} & k_{3,2}\\ k_{1,3} & k_{2,3} & k_{3,3} & k_{1,4}+k_{2,4}+k_{3,4} \end{array}\right]\left\{\begin{array}{l} x_{in1}\\ x_{in2}\\ x_{in3}\\ x_{out} \end{array}\right\}$$

It can be seen from the modeling process that a kinetostatic model of compliant mechanisms with complex topology can be easily established with a low number of elements from the point of view of input/output nodes while using simple steps and concise equations.

#### 3.2.5. Calculating the Kinetostatic Performances of the Compliant Mechanism

Based on this kinetostatic model, the kinetostatic performances, such as input/output stiffness, coupling stiffness, and input/output displacements relations can be derived in concise and explicit matrix forms. Depending on the applied input and output forces, the following two cases will be explained in detail, as follows:

(1) When external loads are applied to input nodes, i.e., *f_ini_*≠0 (*i* = 1,2,3) and *f_out_* = 0

The relationship between the output displacement and input forces can be obtained from Equation (31) in the form of an explicit matrix.

(32) $$x_{out}=\left[\begin{array}{ccc} C_{1} & C_{2} & C_{3} \end{array}\right]\left\{\begin{array}{c} f_{in1}\\ f_{in2}\\ f_{in3} \end{array}\right\}$$

where ***C***_1_, ***C***_2_, and ***C***_3_ are output coupling compliances, which are used to describe the output displacement under the input forces. Moreover, ***C***_1_, ***C***_2_, and ***C***_3_ can also be utilized to analyze the output coupling of the compliant mechanism.

Similarly, the force/displacement relations of input nodes can be expressed as follows:

(33) $$\left\{\begin{array}{c} x_{in1}\\ x_{in2}\\ x_{in3} \end{array}\right\}=\left[\begin{array}{ccc} C_{1,1} & C_{1,2} & C_{1,3}\\ C_{2,1} & C_{2,2} & C_{2,3}\\ C_{3,1} & C_{3,2} & C_{3,3} \end{array}\right]\left\{\begin{array}{c} f_{in1}\\ f_{in2}\\ f_{in3} \end{array}\right\}$$

where ***C****_ij_* (*i* **=** *j*) is the input compliance, which can be utilized to obtain the required input force of the compliant mechanism. This is crucial for the design and selection of actuators. ***C****_ij_* (*i***≠***j*) is the input coupling compliance, which describes the displacement coupling of the input forces of the compliant mechanism. Hence, it can also be utilized to analyze the input coupling of the compliant mechanism.

By combining Equations (32) and (33), the input/output displacements relationship of the compliant mechanism can be expressed as:

(34) $$x_{out}=T\left\{\begin{array}{c} x_{in1}\\ x_{in2}\\ x_{in3} \end{array}\right\}=\left[\begin{array}{ccc} C_{1} & C_{2} & C_{3} \end{array}\right]{\left[\begin{array}{ccc} C_{1,1} & C_{1,2} & C_{1,3}\\ C_{2,1} & C_{2,2} & C_{2,3}\\ C_{3,1} & C_{3,2} & C_{3,3} \end{array}\right]}^{-1}\left\{\begin{array}{c} x_{in1}\\ x_{in2}\\ x_{in3} \end{array}\right\}$$

where ***T*** is a kinematics matrix, which describes the kinematic relationship of the input/output displacements.

Differing from conventional rigid-body mechanisms, the forward kinematic of the compliant mechanism can be easily and accurately derived based on Equation (34) with an explicit matrix form. The motion control and workspace analysis of the compliant mechanism can then be conducted.

(2) When the external load is applied to the output node, i.e., *f_out_*≠0 and *f_ini_* = 0 (*i* = 1,2,3)

The force/displacement relationship of the output node can be derived from the kinetostatic model.

(35) $$x_{out}=K^{-1}f_{out}=Cf_{out}$$

where ***C*** = ***K***^−1^ is the output compliance matrix, which represents the accuracy of this compliant mechanism under the external force applied on the output node.

Similarly, the relationship between the output force and the input displacements can be calculated as follows:

(36) $$\left\{\begin{array}{c} x_{in1}\\ x_{in2}\\ x_{in3} \end{array}\right\}=\left[\begin{array}{l} K_{1}\\ K_{2}\\ K_{3} \end{array}\right]f_{out}$$

where ***K****_i_* (*i* = 1,2,3) can be utilized to analyze the influences of the output force on the input displacements.

## 4. Verification and Discussion

To validate the conciseness and accuracy of the derived modeling method, two typical precision positioning platforms with complex serial–parallel topology are exemplarily conducted. The first case is employed to compare the results of the derived modeling method with those of commercial FEA software Workbench 15.0 (Ansys, Pittsburgh, Pennsylvania, USA). The second case is employed to compare the derived modeling method with an energy-based approach and the matrix displacement approach in previous literature.

### 4.1. First Example

**Figure 8:**
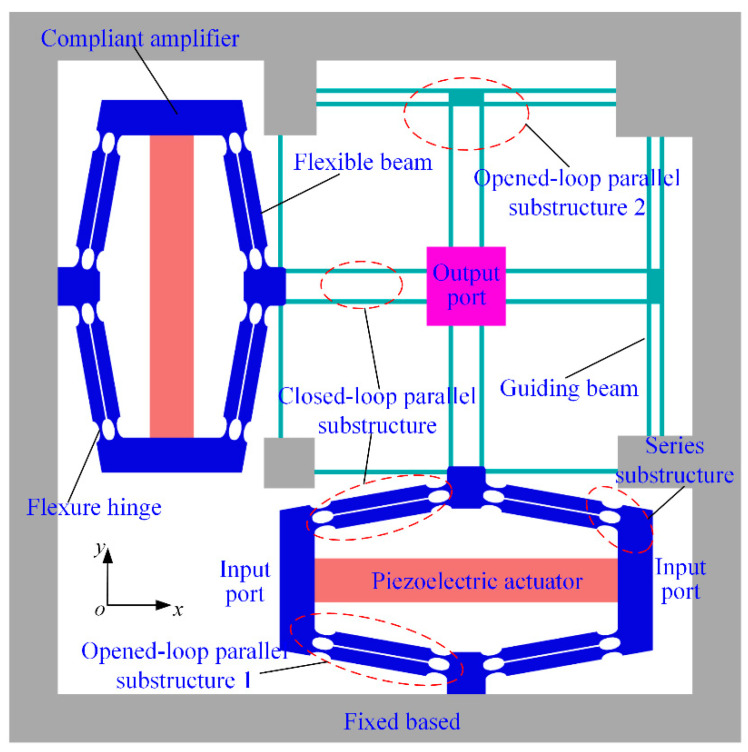
The 2-DOF precision positioning platform.

In the first example, a 2-DOF precision positioning platform is considered to help illustrate and validate the conciseness and accuracy of this modeling method, which is shown in Figure 8. This platform consists of two compliant amplifiers and flexible guiding beams. The motions in the *x*-and *y*-directions are decoupled by flexible guiding beams, and it has similar characteristics in the two directions. The connection relationships of each branch chain can be divided into series substructure, closed-loop parallel substructure, and opened-loop parallel substructure.

**Figure 9:**
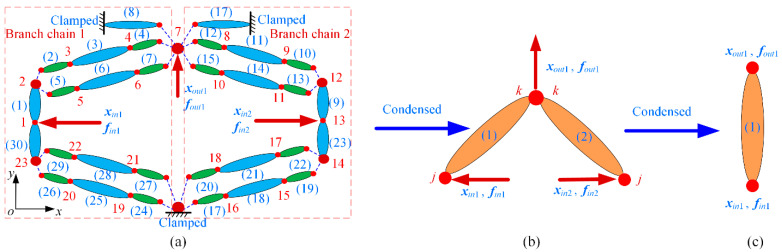
Condensed topology of the compliant amplifier: (**a**) numbering and discretization; (**b**) the equivalent topology; (**c**) the equivalent beam element.

In order to establish the kinetostatic model, the compliant amplifier is first discretized into flexure hinges, flexible beams, and lumped masses. After discretization, it is simplified as a topology including a finite number of elements, which is shown in Figure 9a. The flexible elements are denoted from (1) to (30) and are connected with nodes from 1 to 23. The input forces and output force are numbered as ***f****_in_*_1_, ***f****_in_*_2_, and ***f****_out_*_1_, respectively. According to the connection types of the flexible elements, the transfer matrices of two branch chains from input nodes to the output node is obtained as follows:

(37) $$\left\{\begin{array}{l} T_{ch1}=(Q_{eq1}T_{eq1})(ST_{1})(SQ_{in,1})\\ T_{ch2}=(Q_{eq2}T_{eq2})(ST_{9})(SQ_{in,2}) \end{array}\right.$$

where ***T****_i_*, ***T****_eqi_*, and ***S*** are transfer matrices and can be obtained by Equations (12) and (21). ***Q****_in_*_,1_, ***Q****_eq_*_1_, ***Q****_in_*_,2_, and ***Q****_eq_*_2_ are summation matrices, which can be expressed as:

(38) $$\left\{\begin{array}{l} Q_{in,1}=\left[\begin{array}{cc} E_{6\times 6} & 0_{6\times 6}\\ K_{eq1} & -E_{6\times 6} \end{array}\right],Q_{eq1}=\left[\begin{array}{cc} E_{6\times 6} & 0_{6\times 6}\\ K_{8} & E_{6\times 6} \end{array}\right]\\ Q_{in,2}=\left[\begin{array}{cc} E_{6\times 6} & 0_{6\times 6}\\ K_{eq2} & -E_{6\times 6} \end{array}\right],Q_{eq2}=\left[\begin{array}{cc} E_{6\times 6} & 0_{6\times 6}\\ K_{17} & E_{6\times 6} \end{array}\right] \end{array}\right.$$

where ***K****_eq_*_1_ and ***K****_eq_*_2_ are equivalent stiffness matrices of elements (24)–(30) and elements (17)–(23).

Based on the above analysis, the compliant amplifier with complex serial–parallel topology is condensed into an equivalent topology including only the input nodes and the output node, which is shown in Figure 9b. The force-displacement relationship of node *j* and node *k* of equivalent beam elements can be obtained from Equation (29).

(39) $$\left\{\begin{array}{c} f_{i,j}\\ f_{i,k} \end{array}\right\}=\left[\begin{array}{cc} k_{i,1} & k_{i,2}\\ k_{i,3} & k_{i,4} \end{array}\right]\left\{\begin{array}{c} x_{i,j}\\ x_{i,k} \end{array}\right\}$$

This research takes the input/output nodes as research objects; the force-displacement relationship of the input/output nodes of the compliant amplifier can be calculated based on the equilibrium equations of nodal forces in the form of a matrix.

(40) $$\left\{\begin{array}{l} f_{in1}\\ f_{in2}\\ f_{out1} \end{array}\right\}=\left[\begin{array}{ccc} k_{1,1} & 0 & k_{1,2}\\ 0 & k_{2,1} & k_{2,2}\\ k_{1,3} & k_{2,3} & k_{1,4}+k_{2,4} \end{array}\right]\left\{\begin{array}{l} x_{in1}\\ x_{in2}\\ x_{out1} \end{array}\right\}$$

Due to the symmetrical configuration, the input force ***f****_in_*_2_ can be calculated by the input force ***f****_in_*_1_, ***f****_in_*_2_ = (***−***)***f****_in_*_1_. The force-displacement relationship of the input/output nodes of the compliant amplifier can be further expressed as:

(41) $$\left\{\begin{array}{c} f_{in1}\\ f_{out1} \end{array}\right\}=\left[\begin{array}{cc} kk_{1,1} & kk_{1,2}\\ kk_{1,3} & kk_{1,4} \end{array}\right]\left\{\begin{array}{c} x_{in1}\\ x_{out1} \end{array}\right\}=K_{e1}\left\{\begin{array}{c} x_{in1}\\ x_{out1} \end{array}\right\}$$

where ***kk***_1,1_, ***kk***_1,2_, ***kk***_1,3_, and ***kk***_1,4_ are block sub-matrices of the equivalent beam element, which can be expressed as:

(42) $$\left\{\begin{array}{l} kk_{1,1}=k_{1,1}+k_{4,4},kk_{1,2}=k_{1,2}\\ kk_{1,3}=k_{1,3}-k_{2,3}{\left(k_{2,1}+k_{3,4}\right)}^{-1}kk_{1,1}\\ kk_{1,4}=(k_{1,4}+k_{2,4})-k_{2,3}{\left(k_{2,1}+k_{3,4}\right)}^{-1}(k_{1,2}-k_{2,2}) \end{array}\right.$$

Based on the above analysis, the compliant amplifier is condensed into an equivalent beam element, which is shown in Figure 9c. Equation (41) establishes the two-port kinetostatic model of bridge-type compliant amplifiers and can be further utilized to condense the general precision positioning platform with bridge-type compliant amplifiers.

**Figure 10:**
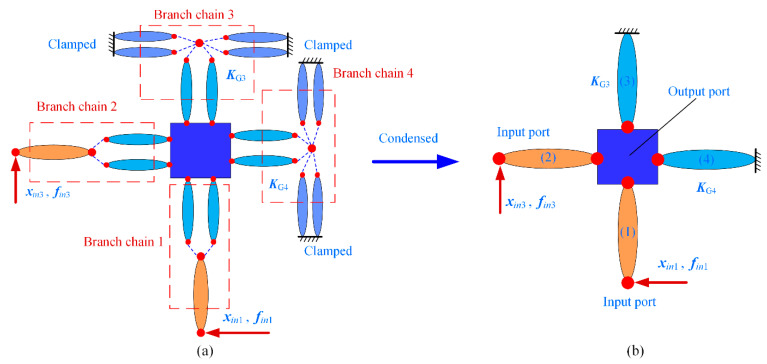
The 2-DOF precision positioning platform: (**a**) numbering and discretization; (**b**) the equivalent topology.

The 2-DOF precision positioning platform is then simplified as an equivalent topology, as shown in Figure 10a. According to the connection types of the flexible elements, the transfer matrices of branch chains 1 and 2 can be expressed as follows:

(43) $$\left\{\begin{array}{l} T_{E1}=T_{b}(ST_{e1})\\ T_{E2}={\left[Tr_{1}\right]}^{T}[T_{E1}][Tr_{1}] \end{array}\right.$$

where ***T****_b_* and ***T****_e_*_1_ are transfer matrices and can be obtained by Equations (12) and (41). ***Tr***_1_ is the rotation transformation matrix, and the rotary angle is 90°.

For the non-driven branch chains, it can be simplified as an equivalent flexible beam based on the compliance matrix approach. The theoretical stiffness matrices of branch chains 3 and 4 are numbered as ***K***_G3_ and ***K***_G4_.

Through the above condensation, the 2-DOF precision positioning platform is further simplified as an equivalent topology, as shown in Figure 10b. The force-displacement relationship of the input/output nodes of the precision positioning platform can then be calculated based on the equilibrium equations of nodal forces in the form of a matrix.

(44) $$\left\{\begin{array}{l} f_{in1}\\ f_{in3}\\ f_{out} \end{array}\right\}=\left[\begin{array}{ccc} k_{E1,1} & 0 & k_{E1,2}\\ 0 & k_{E2,1} & k_{E2,2}\\ k_{E1,3} & k_{E2,3} & k_{E1,4}+k_{E2,4}+K_{G3}+K_{G4} \end{array}\right]\left\{\begin{array}{l} x_{in1}\\ x_{in3}\\ x_{out} \end{array}\right\}$$

The relationships between the output displacement, input displacements, and input forces of the precision positioning platform can be obtained from Equation (40) under the output force ***f****_out_* = **0**.

(45) $$\left\{\begin{array}{l} x_{out}=\left[\begin{array}{cc} C_{1} & C_{2} \end{array}\right]\left\{\begin{array}{l} f_{in1}\\ f_{in3} \end{array}\right\}\\ \left\{\begin{array}{l} x_{in1}\\ x_{in3} \end{array}\right\}=\left[\begin{array}{cc} C_{1,1} & C_{1,2}\\ C_{2,1} & C_{2,2} \end{array}\right]\left\{\begin{array}{l} f_{in1}\\ f_{in3} \end{array}\right\}\\ x_{out}=\left[\begin{array}{cc} C_{1} & C_{2} \end{array}\right]{\left[\begin{array}{cc} C_{1,1} & C_{1,2}\\ C_{2,1} & C_{2,2} \end{array}\right]}^{-1}\left\{\begin{array}{l} f_{in1}\\ f_{in3} \end{array}\right\} \end{array}\right.$$

where ***C***_1_ and ***C***_2_ are the output coupling compliances. ***C****_ij_* (*i* **=** *j*) is the input compliance and ***C****_ij_* (*i* **≠** *j*) is the input coupling compliance. ***f****_in_*_1_ = [-*f*_pzt_, 0, 0, 0, 0, 0]^T^ and ***f****_in_*_3_ = [*f*_pzt_, 0, 0, 0, 0, 0]^T^ denote the input forces from the piezoelectric actuators.

The static performances of the precision positioning platform, such as input stiffness *K_in_* and displacement amplification ratio *R*, can be expressed as:

(46) $$K_{in}=-\frac{f_{\mathrm{pzt}}}{x_{in1(x)}}=\frac{f_{\mathrm{pzt}}}{x_{in3(y)}},\begin{array}{cc} & R=\frac{x_{out(y)}}{2\cdot x_{in1(x)}} \end{array}$$

where ***x****_out_*_(*y*)_, ***x****_in_*_3(*y*)_, and ***x****_in_*_1(*x*)_ are the nodal displacements in the *y*-direction and the *x*-direction.

**Figure 11:**
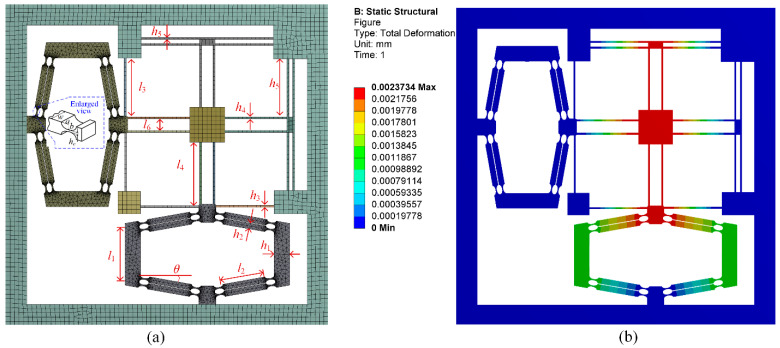
Finite element analysis. (**a**) geometric parameters; (**b**) result of one simulation set.

**Figure 12:**
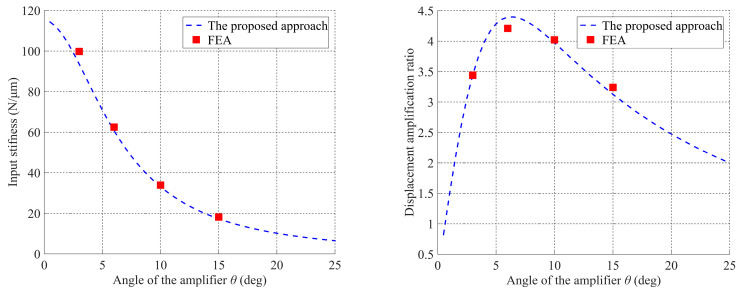
Simulation results of static performances.

For comparison, the precision positioning platform is analyzed by employing Workbench 15.0. The geometric parameters of this platform are defined in Figure 11, and the specific values are listed in Table 1. When an actuating force of *f*_pzt_ = 10 N along *x*-direction is applied to the input nodes, the output displacement in the *y*-direction and input displacements in the *x*-direction can be obtained from the Workbench 15.0. The finite element analysis results of input stiffness and displacement amplification ratio can then be calculated based on Equation (46). As shown in Figure 12, four sets of simulation results for input stiffness and displacement amplification ratio are carried out by selecting a different angle *θ* of the bridge-type compliant amplifier, while keeping other parameters unchanged. Quantitative comparison between the derived kinetostatic model and commercial FEA software Workbench 15.0 is shown in Table 2.

From the calculated results shown in Figure 12, it can be observed that the theoretical calculation results of the derived kinetostatic model can match well with those of the commercial software Workbench 15.0. At the same time, the comparison results listed in Table 2 show that relative errors of the theoretical model in regard to the commercial software Workbench 15.0 are less than 6.2% regarding both the input stiffness and displacement amplification ratio. These results also indicate that the derived modeling approach can predict the kinetostatic performances of compliant mechanisms with satisfactory accuracy.

### 4.2. Second Example

**Figure 13:**
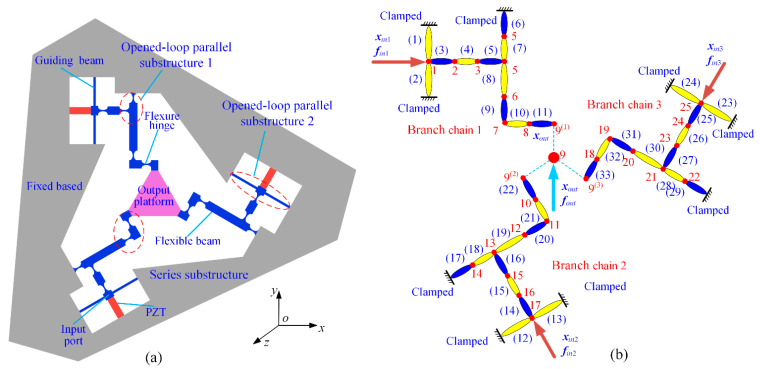
The 3-DOF precision positioning platform: (**a**) schematic; (**b**) discretization and numbering.

In the second example, the 3-DOF precision positioning platform in references [24,35], which is shown in Figure 13, is employed to validate the proposed modeling method by comparing the kinematic and static performances of this positioning platform with four approaches: (a) the proposed modeling approach; (b) the matrix displacement approach; (c) the energy-based approach; (d) the finite element analysis by the commercial software Workbench 15.0. For comparability, the material and geometric parameters of this platform are consistent with those in references [24,35]. The 3-DOF precision positioning platform is then discretized and numbered. It can be observed that this precision positioning platform is composed of 33 elements and 25 nodes. The input forces and output force are, respectively, numbered as ***f****_in_*_1_, ***f****_in_*_2_, ***f****_in_*_3_, and ***f****_out_*. Considering the symmetry configuration of the platform, only one branch chain is employed to illuminate the proposed modeling approach. According to the connection types of flexible elements, the transfer matrix of branch chain (1) can be calculated as:

(47) $$T_{ch1}=(T_{11})(ST_{10})(ST_{9})(ST_{8})(SQ_{5}T_{5})(ST_{4})(ST_{3})(SQ_{in,1})$$

where ***T****_i_* and ***S*** are transfer matrices and given by Equations (12) and (21). ***Q****_in_*_,1_ and ***Q***_5_ are summation matrices, which can be expressed as:

(48) $$Q_{in,1}=\left[\begin{array}{cc} E_{6\times 6} & 0_{6\times 6}\\ K_{1}+K_{2} & -E_{6\times 6} \end{array}\right],Q_{5}=\left[\begin{array}{cc} E_{6\times 6} & 0_{6\times 6}\\ K_{eq} & E_{6\times 6} \end{array}\right]$$

By performing rotation transformation, the transfer matrices of branch chains (2) and (3) can be expressed as:

(49) $$\left\{\begin{array}{l} \left[T_{ch2}]={\left[Tr_{2}\right]}^{T}[T_{ch1}][Tr_{2}\right]\\ \left[T_{ch3}]={\left[Tr_{3}\right]}^{T}[T_{ch1}][Tr_{3}\right] \end{array}\right.$$

where ***Tr***_2_ and ***Tr***_3_ are rotation transformation matrices. The rotary angles of ***Tr***_2_ and ***Tr***_3_ are 120° and 240°, respectively.

**Figure 14:**
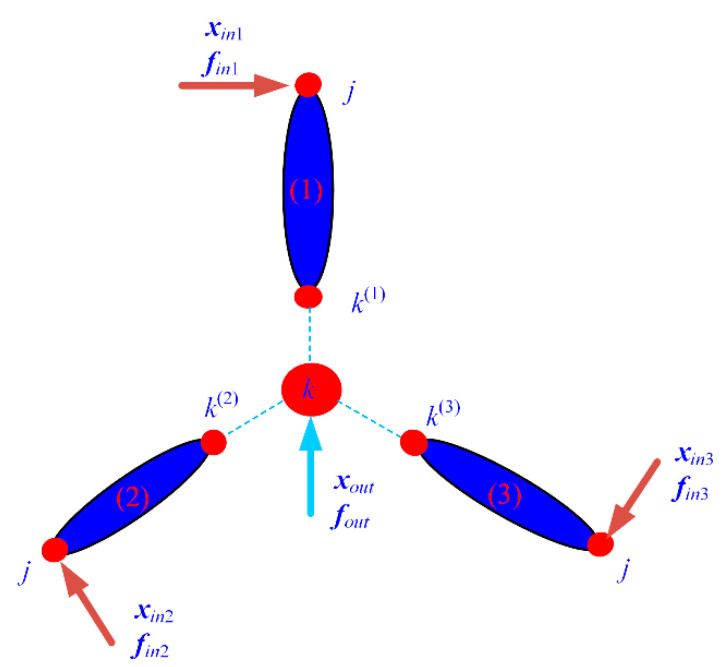
The equivalent topology of this platform.

Through the above analysis, the 3-DOF precision positioning platform in Figure 13 can be simplified as an equivalent topology only including input nodes and output node, which is shown in Figure 14. This research takes input/output nodes as the research objects and calculates the force-displacement relationship of the input/output node of the precision positioning platform based on the principle of equilibrium of nodal forces in the form of a matrix.

(50) $$\left\{\begin{array}{l} f_{in1}\\ f_{in2}\\ f_{in3}\\ f_{out} \end{array}\right\}=\left[\begin{array}{cccc} k_{in1,1} & 0 & 0 & k_{in1,2}\\ 0 & k_{in2,1} & 0 & k_{in2,2}\\ 0 & 0 & k_{in3,1} & k_{in3,2}\\ k_{in1,3} & k_{in2,3} & k_{in3,3} & k_{in1,4}+k_{in2,4}+k_{in3,4} \end{array}\right]\left\{\begin{array}{l} x_{in1}\\ x_{in2}\\ x_{in3}\\ x_{out} \end{array}\right\}$$

According to the force-displacement relationship of the 3-DOF precision positioning platform, the kinetostatic performances, such as input/output stiffness, coupling stiffness, and input/output displacements relations can be derived by Equations (32)–(36).

**Figure 15:**
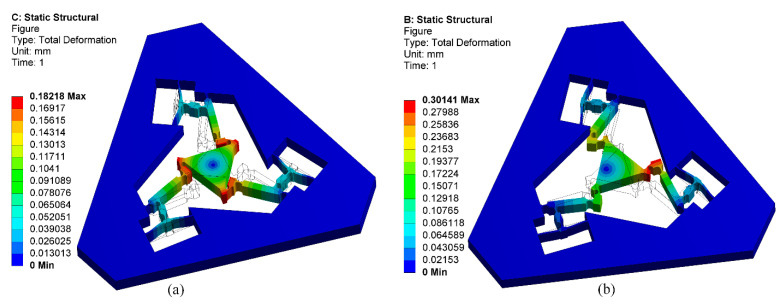
Finite element results. (**a**) ***f****_in_*_1_ = ***f****_in_*_2_ = ***f***_in3_ = 100 N; (**b**) ***f****_out_* = [0 N, 50 N, 0 N, 0 N·m, 0 N·m, 1 N·m].

For finite element analysis, the 3-DOF precision positioning platform is modeled through SolidWorks, and then the kinetostatic performances can be calculated with Workbench 15.0. The material and geometric parameters of the platform are obtained from references [24,35]. In order to calculate the kinetostatic performances in Workbench 15.0, two sets of load conditions of ***f****_in_*_1_ = ***f****_in_*_2_ = ***f***_in3_ = 100 N and ***f****_out_* = [0 N, 50 N, 0 N, 0 N·m, 0 N·m, 1 N·m] are, respectively, applied on the input nodes and the output node of the precision positioning platform. The input displacements and output displacement results obtained by commercial FEA software Workbench 15.0 under two conditions are shown in Figure 15. Meanwhile, the detailed comparative results with four different approaches are listed in Table 3 and Table 4.

It can be observed that the theoretical calculation results of the proposed modeling approach and the energy-based approach in reference [35] are closer to those of the commercial FEA software Workbench 15.0 than the matrix displacement approach in reference [24]. However, the proposed modeling approach can describe the kinetostatic performances of the precision positioning platform with a lower number of elements than the energy-based approach in reference [35]. The maximum deviation between the proposed modeling approach and commercial FEA software is less than 4.3% under the input force of ***f****_in_*_1_ = ***f****_in_*_2_ = ***f****_in_*_3_ = 100 N and less than 2.8% under the output force of ***f****_out_* = [0 N, 50 N, 0 N, 0 N·m, 0 N·m, 1 N·m] for the input/output displacements. However, the number of elements in the finite element analysis is one hundred and twenty thousand, while the proposed modeling approach only needs three elements. To sum up, the proposed modeling approach can accurately calculate the kinetostatic performances of compliant mechanisms with a low number of elements by using simple steps and concise equations.

## 5. Conclusions

This paper develops a substructure condensed approach for the design and analysis of compliant mechanisms with complex serial–parallel topology. The element transfer matrix of the common flexible element is established by employing the energy conservation law. Based on the equilibrium equations of nodal forces and the transfer matrix approach, this paper calculates the general kinetostatic model of compliant mechanisms with a low number of elements. The kinematic and static performances, for instance, input stiffness, output stiffness, coupling stiffness, and input/output displacement relations with concise and explicit forms, can be easily obtained from the proposed kinetostatic model. The proposed modeling approach is verified by comparing this approach with existing modeling approaches and finite element analysis. The comparison results validate the conciseness and efficiency of this approach. The substructure condensed approach proposed in this paper can be further utilized in the optimization design and motion control for complex compliant mechanisms.

## Figures and Tables

**Table 1:** Geometric and material parameters of the platform.

Parameters	Values	Parameters	Values	Parameters	Values
*l*_1_ (mm)	14.0	*l*_4_ (mm)	16	*b* (mm)	0.6
*h*_1_ (mm)	4.0	*h*_4_ (mm)	0.5	*h*_e_ (mm)	0.3
*l*_2_ (mm)	11.0	*l*_5_ (mm)	15	*w* (mm)	10.0
*h*_2_ (mm)	1.5	*h*_5_ (mm)	0.5	*θ* (deg)	10.0
*l*_3_ (mm)	15.0	*l*_6_ (mm)	3.0	*E* (GPa)	200
*h*_3_ (mm)	0.5	*a* (mm)	1.2	*G* (GPa)	77.64

**Table 2:** Comparison results of static performances.

Angle *θ* (deg)	*K_in_* (N/μm)			*R*		
	The Proposed Approach	FEA	Error (%)	The Proposed Approach	FEA	Error (%)
3	93.61	99.80	6.20	3.44	3.24	6.17
6	60.72	62.50	2.85	4.39	4.21	4.28
10	33.07	33.90	2.45	3.97	4.02	1.24
15	17.30	18.18	4.84	3.12	3.05	2.30

**Table 3:** The comparative results of the four different methods under the input force of ***f****_in_*_1_ = ***f****_in_*_2_ = ***f***_in3_ = 100 N.

Approaches	*f_in_*_1_ = *f_in_*_2_ = *f_in_*_3_ = 100 N								Elements
	*x_in_*_1_ (μm)	*x_in_*_2_ (μm)		*x_in_*_3_ (μm)		*x_out_* (μm/rad)			
	*u*	*u*	*v*	*u*	*v*	*u*	*v*	*θ*	
FEA	42.5	−21.4	36.9	−21.4	−36.9	0	0	0.0116	120,000
Reference [24]	46.3	−23.1	40.1	−23.2	−40.1	0	0	0.0130	9
Reference [35]	41.1	−20.5	35.6	−20.5	35.6	0	0	0.0114	33
The proposed approach	41.1	−20.5	35.6	−20.6	35.5	0	0	0.0114	3
Error	3.1%	4.2%	3.5%	4.2%	3.5%	0	0	1.7%	

**Table 4:** The input/output displacements under the output force of ***f****_out_* = [0 N, 50 N, 0 N, 0 N·m, 0 N·m, 1 N·m].

Approaches	*f_out_* = [0 N, 50 N, 0 N, 0 N·m, 0 N·m, 1 N·m]								Elements
	*x_in_*_1_ (μm)	*x_in_*_2_ (μm)		*x_in_*_3_ (μm)		*x_out_* (μm/rad)			
	*u*	*u*	*v*	*u*	*v*	*u*	*v*	*θ*	
FEA	−47.5	10.7	−18.1	22.1	39.4	0	68.4	0.0149	120,000
The proposed approach	−47.8	10.5	−18.3	22.7	39.2	0	68.7	0.0150	3
Error	0.6%	1.9%	1.1%	2.7%	0.5%	0	0.4%	0.7%	

## Data Availability

Not applicable.

## References

[1] Howell L.L. (2001). Compliant Mechanisms.

[2] Chen G., Han Q., Jin K. (2019). A fully compliant tristable mechanism employing both tensural and compresural segments. J. Mech. Robot..

[3] Valentini P.P., Pennestrì E. (2018). Compliant four-bar linkage synthesis with second-order flexure hinge approximation. Mech. Mach. Theory.

[4] Valentini P.P., Cirelli M., Pennestrì E. (2019). Second-order approximation pseudo-rigid model of flexure hinge with parabolic variable thickness. Mech. Mach. Theory.

[5] Liao S., Ding B., Li Y. (2022). Design, assembly, and simulation of flexure-based modular micro-positioning stages. Machines.

[6] Xiao X., Xi R., Li Y., Tang Y., Ding B., Ren H., Meng M. (2021). Design and control of a novel electromagnetic actuated 3-DoFs micropositioner. Microsyst. Technol..

[7] Yang M., Du Z., Chen F., Dong W., Zhang D. (2017). Kinetostatic modelling of a 3-PRR planar compliant parallel manipulator with flexure pivots. Precis. Eng..

[8] Shao Z., Wu S., Wu J., Fu H. (2017). A novel 5-DOF high-precision compliant parallel mechanism for large-aperture grating tiling. Mech. Sci..

[9] Zhu Z., To S., Zhang S. (2015). Theoretical and experimental investigation on the novel end-fly-cutting-servo diamond machining of hierarchical micro-nanostructures. Int. J. Mach. Tools Manuf..

[10] Wei Y., Xu Q. (2022). Design of a new passive end-effector based on constant-force mechanism for robotic polishing. Robot. Comput.-Integr. Manuf..

[11] Ding B., Zhao J., Li Y. (2021). Design of a spatial constant-force end-effector for polishing/deburring operations. Int. J. Adv. Manuf. Technol..

[12] Ding B., Li X., Li Y. (2022). Configuration design and experimental verification of a variable constant-force compliant mechanism. Robotica.

[13] Ursi P., Rossi A., Botta F., Belfiore N.P. (2022). Analytical Modeling of a New Compliant Microsystem for Atherectomy Operations. Micromachines.

[14] Shi H., Su H.J., Dagalakis N. (2014). A stiffness model for control and analysis of a MEMS hexapod nanopositioner. Mech. Mach. Theory.

[15] Su H.J., Shi H., Yu J. (2012). A symbolic formulation for analytical compliance analysis and synthesis of flexure mechanisms. J. Mech. Des. Trans. ASME.

[16] Ling M., Howell L.L., Cao J., Chen G. (2019). Kinetostatic and dynamic modeling of flexure-based compliant mechanisms: A survey. Appl. Mech. Rev..

[17] Ling M., Cao J., Jiang Z., Lin J. (2017). Modular kinematics and statics modeling for precision positioning stage. Mech. Mach. Theory.

[18] Ling M., Cao J., Zeng M., Lin J., Inman D.J. (2016). Enhanced mathematical modeling of the displacement amplification ratio for piezoelectric compliant mechanisms. Smart Mater. Struct..

[19] Lobontiu N., Garcia E. (2003). Analytical model of displacement amplification and stiffness optimization for a class of flexure-based compliant mechanisms. Comput. Struct..

[20] Chen W., Zhang X., Li H., Wei J., Fatikow S. (2017). Nonlinear analysis and optimal design of a novel piezoelectric-driven compliant microgripper. Mech. Mach. Theory.

[21] Zhu Z., To S., le Zhu W., Li Y., Huang P. (2018). Optimum Design of a Piezo-Actuated Triaxial Compliant Mechanism for Nanocutting. IEEE Trans. Ind. Electron..

[22] Li Y., Huang J., Tang H. (2012). A compliant parallel XY micromotion stage with complete kinematic decoupling. IEEE Trans. Autom. Sci. Eng..

[23] Jiang Y., Li T., Wang L. (2015). Stiffness modeling of compliant parallel mechanisms and applications in the performance analysis of a decoupled parallel compliant stage. Rev. Sci. Instrum..

[24] Ling M., Cao J., Howell L.L., Zeng M. (2018). Kinetostatic modeling of complex compliant mechanisms with serial-parallel substructures: A semi-analytical matrix displacement method. Mech. Mach. Theory.

[25] Ling M., Cao J., Pehrson N. (2019). Kinetostatic and dynamic analyses of planar compliant mechanisms via a two-port dynamic stiffness model. Precis. Eng..

[26] Zhu W., Rui X.T. (2017). Modeling of a three degrees of freedom piezo-actuated mechanism. Smart Mater. Struct..

[27] Ma H.W., Yao S.M., Wang L.Q., Zhong Z. (2006). Analysis of the displacement amplification ratio of bridge-type flexure hinge. Sens. Actuators A Phys..

[28] Lobontiu N., Paine J.S.N., Garcia E., Goldfarb M. (2001). Corner-filleted flexure hinges. J. Mech. Des. Trans. ASME.

[29] Shi R.C., Dong W., Du Z.J. (2013). Design methodology and performance analysis of application-oriented flexure hinges. Rev. Sci. Instrum..

[30] Pham H.H., Chen I.M. (2005). Stiffness modeling of flexure parallel mechanism. Precis. Eng..

[31] Li Y., Xu Q. (2012). Design and robust repetitive control of a new parallel-kinematic XY piezostage for micro-nano manipulation. IEEE/ASME Trans. Mechatron..

[32] Li Y., Xu Q. (2010). Development and assessment of a novel decoupled XY parallel micropositioning platform. IEEE/ASME Trans. Mechatron..

[33] Noveanu S., Lobontiu N., Lazaro J., Mandru D. (2015). Substructure compliance matrix model of planar branched flexure-hinge mechanisms: Design, testing and characterization of a gripper. Mech. Mach. Theory.

[34] Lobontiu N. (2014). Compliance-based matrix method for modeling the quasi-static response of planar serial flexure-hinge mechanisms. Precis. Eng..

[35] Wu S., Shao Z., Su H., Fu H. (2019). An energy-based approach for kinetostatic modeling of general compliant mechanisms. Mech. Mach. Theory.

[36] Chen G., Liu X., Gao H., Jia J. (2009). A generalized model for conic flexure hinges. Rev. Sci. Instrum..

[37] Chen G., Shao X., Huang X. (2008). A new generalized model for elliptical arc flexure hinges. Rev. Sci. Instrum..

[38] Ling M., Cao J., Jiang Z., Lin J. (2018). A semi-analytical modeling method for the static and dynamic analysis of complex compliant mechanism. Precis. Eng..

